# The immune response of inbred laboratory mice to *Litomosoides sigmodontis*: A route to discovery in myeloid cell biology

**DOI:** 10.1111/pim.12708

**Published:** 2020-03-21

**Authors:** Conor M. Finlay, Judith E. Allen

**Affiliations:** ^1^ Lydia Becker Institute for Immunology & Infection Faculty of Biology Medicine & Health, Manchester Academic Health Science Centre University of Manchester Manchester UK

**Keywords:** helminths, *Litomosoides sigmodontis*, macrophages, Th2 cells

## Abstract

*Litomosoides sigmodontis* is the only filarial nematode where the full life cycle, from larval delivery to the skin through to circulating microfilaria, can be completed in immunocompetent laboratory mice. It is thus an invaluable tool for the study of filariasis. It has been used for the study of novel anti‐helminthic therapeutics, the development of vaccines against filariasis, the development of immunomodulatory drugs for the treatment of inflammatory disease and the study of basic immune responses to filarial nematodes. This review will focus on the latter and aims to summarize how the *L sigmodontis* model has advanced our basic understanding of immune responses to helminths, led to major discoveries in macrophage biology and provided new insights into the immunological functions of the pleural cavity. Finally, and most importantly *L sigmodontis* represents a suitable platform to study how host genotype affects immune responses, with the potential for further discovery in myeloid cell biology and beyond.

## 1 INTRODUCTION

Filarial nematodes are thread‐like tissue‐dwelling roundworms that are transmitted by arthropod vectors and infect over 120 million people worldwide.^1^, ^2^, ^3^ The most well‐known of human filarial nematodes, *Wuchereria bancrofti* and *Brugia malayi,* reside in the lymphatics and are thus referred to as lymphatic filaria. The other major filarial nematode of human consequence, *Onchocerca volvulus,* forms nodules in subcutaneous tissue. However, all filaria including the focus of this review, *Litomosoides sigmodontis*, migrate through the lymphatics as part of their life cycle.^4^ A clinically important feature of filarial life cycles is that sexually mature female worms produce microfilaria (mF) a larval form that are acquired by the arthropod vector via the blood or skin.^5^ Infections which lead to detectable circulating microfilaraemia in the mammalian host are said to be patent.

Filarial nematodes are best known as the causative agents of the disfiguring and disabling lymphatic filariasis (lymphoedema, hydrocele and elephantiasis) and onchocerciasis (river blindness/sowda), as well as the less severe loasis.^1^, ^6^, ^7^ Clinical symptoms are generally a consequence of damage to lymphatic vessels or chronic immunopathology in infected tissue.^6^ Infected individuals also suffer from periodic attacks of fever.^6^ However, the simplistic causal relationship between filarial nematodes and immunopathology fails to capture the reality of infection on a population level.^8^ In areas endemic for filarial diseases, there are a variety of outcomes to infection. Some ‘resistant’ individuals clear worms prior to the onset of patency, while others develop long‐lasting infections with or without microfilaraemia. Asymptomatic (or subclinical) infection is typically associated with high levels of circulating microfilariae. Progression to the characteristic clinical pathologies is also highly variable. In lymphatic filariasis, pathology can occur in the presence or absence of detectable parasitaemia. In onchocerciasis, pathology is more common in infected individuals which do not have mF.^1^, ^6^, ^8^, ^9^, ^10^ These divergent clinical outcomes highlight the importance of understanding how host genotype impacts upon the immune response to filarial nematodes.

While *L sigmodontis* infection of mice does not result in the characteristic pathology seen in humans, it does model some of the diversity of immune responses. Different inbred strains of mice exhibit distinct susceptibility/resistance to infection, particularly in relation to mF, along with remarkably different immune responses to the parasite. Here, we wish to provide a cell‐by‐cell resource that summarizes what we know about the immune response to *L sigmodontis* infection with respect to the divergent nature of immune responses in susceptible and resistant hosts. We also highlight the power of this strain comparison for new discovery in filariasis, type 2 immune responses and myeloid biology.

## 2 L sigmodontis

*L sigmodontis* is a filarial nematode of the Onchocercidae family first isolated from the cotton rat *Sigmodon hispidus*.^11^ The site of infection is the pleural cavity which resembles the relatively rare human diseases caused by *Mansonella ozzardi* and *M perstans*.^3^ *L sigmodontis* is transmitted to the primary host via an arthropod vector, the tropical mite *Ornithonyssus bacoti,* which acts as the intermediate host and a reservoir for infective *L sigmodontis* L3 larvae. The life cycle and maintenance of *L sigmodontis* in a laboratory setting have been described in detail elsewhere.^12^ For experiments with laboratory mice, infection can be achieved by allowing infected mites to feed naturally, with L3 larvae entering the skin when the mite performs a blood meal (natural infection). Alternatively, a known number of L3 larvae can be isolated from mites and injected directly into mice (subcutaneous infection).^13^ Although natural infection includes the activation of innate immune responses in the skin that follow mite feeding, subcutaneous infection allows the infective dose to be known, and the immunological findings from laboratories that use the different routes have not fundamentally differed.

Many L3 larvae are destroyed in the skin.^14^, ^15^ Surviving larvae forcibly enter lymphatic vessels^15^, ^16^ and migrate to the pleural cavity by about day 4 post‐infection (p.i.). The exact route the worms take on their way to the pleural cavity is not well understood but may involve translocation through the lung.^17^ At around day 8, the worm undergoes a moult to become an L4 larva and another final moult at circa day 28‐30 to become an adult. However, it takes another 30 days for the worms to become sexually mature and produce mF. It remains the only filarial nematode in which infection with larvae leads to circulating mF in the immunocompetent murine host.

## 3 THE SITE OF INFECTION: THE PLEURAL CAVITY

A major advantage of the *L sigmodontis* model is that the site of infection is simple in terms of tissue architecture. Isolation of worms and immune cells is performed by lavage of the pleural cavity, without the need for destructive tissue homogenization or digestion.

**FIGURE 1:**
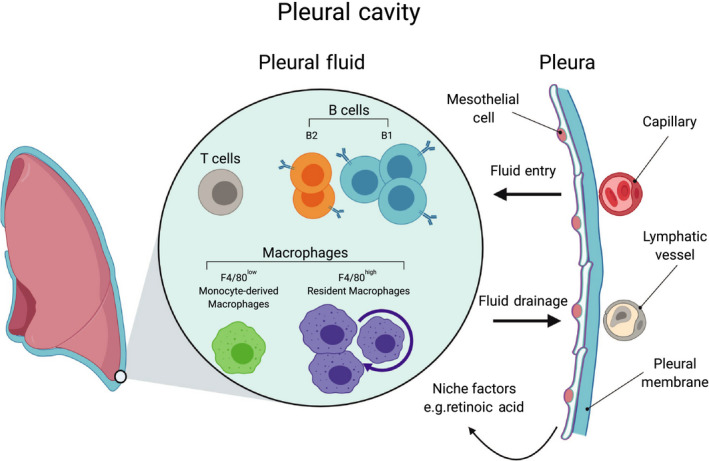
The Pleural Cavity. The pleural cavity is a fluid‐filled space created by a two‐membrane (pleura) structure that lines the lung and chest wall. The pleural fluid supports breathing by providing lubrication and by allowing close apposition of the inner pleura, covering the lung, with the outer pleura, covering the chest wall. Pleural fluid enters the cavity via the intercostal arteries and is drained by the lymphatics. The pleural fluid is cell dense with CD45^+^ immune cells. In mice, these include populations of B cells (B1 and B2 cells), macrophages (F480^high^ and F4/80^low^) and T cells with smaller numbers of mast cells and dendritic cells

The pleura is a serous membrane with a two‐layer membrane structure folded back on itself made up of a layer of mesothelial cells (the mesothelium).^18^ Lining the lung is the visceral membrane, and lining the chest wall and diaphragm is the parietal membrane.^19^ The space between the two membranes is only in potential a cavity. In reality, it is a thin layer of fluid kept at negative pressure which allows the lungs to remain ‘attached’ to the parietal membrane when the chest expands, thus catering for lung inflation.^19^, ^20^ The pleural fluid contains lysozyme, antibody, complement and proteins such as surfactants, that reduce the friction of breathing.^19^, ^20^ Many of these factors, including clotting factors and complement, are locally produced rather than entering the cavity via the serum. Pleural fluid enters via capillaries lining the parietal membrane and is drained via lymphatics within the cavity (Figure 1).^20^ The pleural fluid is highly cellular and due to breathing, is in constant motion. The cells of the pleural fluid are almost exclusively CD45^+^ immune cells. In naïve mice, these are comprised of, in order of abundance, B cells (B1 then B2 cells), macrophages (F4/80^high^ resident macrophages then F4/80^low^ monocyte‐derived macrophages), T cells, and small numbers of dendritic cells, NK cells and mast cells (Figure 1) (CM Finlay, personal observations).

Infection with *L sigmodontis* results in oedema of the pleural cavity (pleural effusion) which is accompanied by the expansion of immune cells in the fluid through proliferation or recruitment. Eosinophils and neutrophils are absent from the pleural fluid of naïve mice but are recruited during infection. Hence, for the duration of infection, worms remain in immediate proximity to the immune cells of the pleural fluid. *L sigmodontis,* like other filaria, perform continuous whip like movements known as the ‘filarial dance’ that may act as a method to prevent the attachment of such immune cells.^21^

## 4 STRAIN DEPENDENCY

**FIGURE 2:**
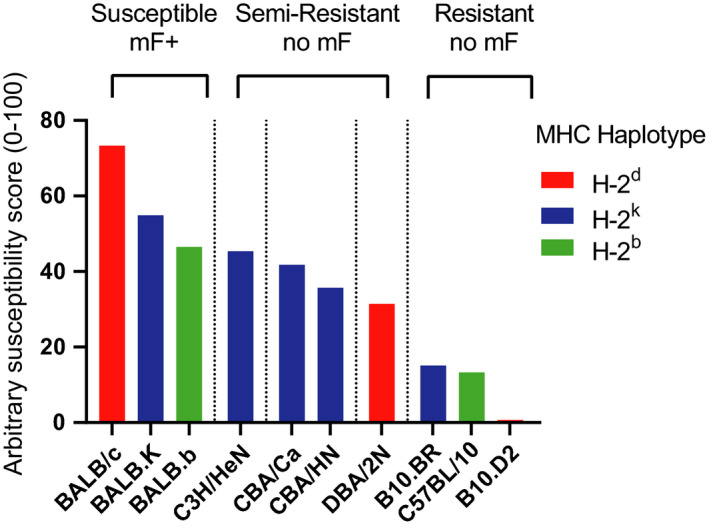
Resistance to *L sigmodontis* infection varies across host genotype. Data from Tables 2 (readouts of *L sigmodontis* infection in ten strains of mice) and III (readouts of morphology of *L sigmodontis* worms taken from ten strains of mice) from Petit et al^23^ were reanalysed to create a relative susceptibility score. Only data for male mice were included. The following readouts were collated and weighted as follows: mF/10 μL of blood was scaled 0‐100 with 100 being given to the strain with the highest mF numbers (weighted 2), percentage mF^+^ (weighted 2), percentage worm^+^ (weighted 1), percentage localization of worms in the pleural cavity (weighted 0.5), number of worms recovered as a percentage of injected L3 larvae (weighted 1.5), length worms scaled to longest worm recovered from any strain (weighted 0.25 each for male and female worms), width of worms scaled to widest worm recovered (weighted 0.25 each for male and female worms), uterine egg score for recovered worms (weighted 0.25), divided egg score for recovered worms (weighted 0.25), percentage of worms with internal mF (weighted 1) and mF score for recovered worms (weighted 0.5). Weighted scores were added together to give a total possible score of 1000, and this number was divided by 100 to give scores shown in the graph above. Bar colour represents MHC haplotype for each strain

*L sigmodontis* was first used to infect ‘albino’ mice in 1946, but it was not widely used as a murine model until the early 1990s.^22^ The establishment of *L sigmodontis* as a mouse model to study filarial nematode infection and strain differences owes much to the pioneering work of Odile Bain's laboratory at Muséum National d'Histoire Naturelle, Paris. Most notably, their paper from 1992 which investigated the susceptibility of various strains of mice to *L sigmodontis* using a number of quantitative readouts of worm health and sexual maturity at a later stage of infection.^23^ We have compiled and reanalysed the parasitology data from that study to provide an overview of relative susceptibility across host genotype (Figure 2. the method of analysis is described in the figure legend). Strains of mice can be subdivided into mice which are susceptible and microfilaraemic (BALB sublines), semi‐resistant (C3H, CBA and DBA), or resistant (C57BL/10 sublines) (Figure 2).

Notably, resistance does not segregate with MHC haplotype as all BALB mice despite MHC subtype (H‐2^c^, H‐2^k^ or H‐2^d^) are poor controllers of infection while C57BL/10 (H‐2^b^) or B10.D2 (H‐2^d^) are highly resistant.^23^ BALB/c v B10.D2 are the most divergent in terms of susceptibility/resistance yet share the same MHC haplotype (H‐2^d^) (Figure 2).^5^, ^23^ The more widely available C57BL/6 strain shares the resistance phenotype seen in C57BL/10 sublines.^24^, ^25^, ^26^ The C57BL/6‐resistant vs BALB/c‐susceptible comparison is now the established model for strain comparison in *L sigmodontis.*

**FIGURE 3:**
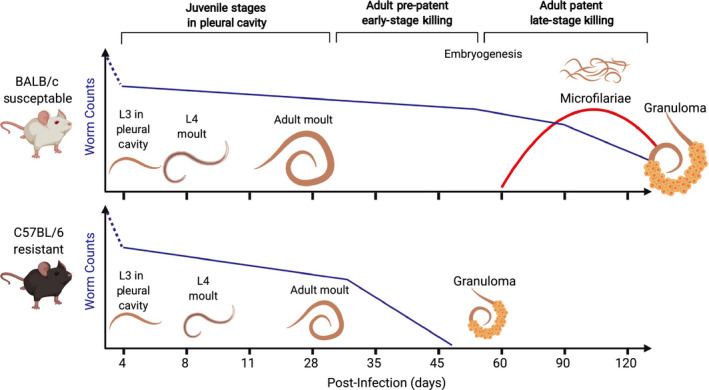
Timeline of experimental *L sigmodontis* infection. Mice are infected by allowing *L sigmodontis‐*infected *O bacoti* mites to blood feed on mice (natural infection) or by direct injection of a known number of *L sigmodontis* L3 larvae subcutaneously. Most L3 larvae are killed in the skin but those that do survive enter the lymphatics and typically appear in the pleural cavity by day 4 p.i. In the pleural cavity, *L sigmodontis* undergo a moult to an L4 larva around day 8 p.i. and another moult to adulthood around day 28‐30 p.i. Few worms are killed between arrival and adult moult in either strain. At day 35 p.i., worms are significantly larger in BALB/c‐susceptible mice than in resistant C57BL/6 mice. At this time point, worms begin to die in C57BL/6 mice whereas they continue to survive in BALB/c mice. Between day 35 and day 60 p.i. in BALB/c mice, worms become sexually mature, mate and undergo embryogenesis. Beginning around day 55 p.i., female worms in BALB/c mice produce mF which are detectable in the blood stream. Worms are gradually killed from day 80 onwards, and blood microfilaraemia is correspondingly reduced

Only about one third or less L3 survive the 4 day journey from the skin to the pleural cavity.^24^, ^26^ Prior to day 20 p.i., L4 larvae are significantly larger in susceptible BALB/c mice than C57BL/6 mice^26^ with a small difference in worm number (Figure 3).^24^ Despite these differences, there is little observable worm killing in the pleural cavity in either strain before the adult moult around day 28‐30 p.i. (Figure 3).^24^, ^26^ Later in infection, obvious differences emerge. C57BL/6 mice begin to kill worms around day 35 p.i., and from day 40 p.i. onwards, dead and granulomatous worms are readily detectable in the pleural lavage. In contrast, worms in BALB/c mice continue to grow, undergo embryogenesis and by day 56 p.i. start to produce mF which are detectable in the bloodstream (Figure 3).^24^, ^26^

Patency has a strong sexual dimorphism.^23^, ^24^ Depending on host sex, approximately 20% (male) to 70% (female) of BALB/c mice will develop patent infection. The presence of mF^‐^ individuals and the wide range in mF numbers is reflective of human filarial infections. Female mice harbour more adult worms than males and are over four times more prone to be mF^+^ than males, independent of the number of adult worms borne.^24^ Nonetheless, in all BALB/c mice, worms are eventually killed between days 90 and 120 p.i. Thus, while they are represented as ‘susceptible’, BALB/c mice are more accurately semi‐susceptible in comparison with the cotton rat or the Mongolian jird (*Meriones unguiculatus)* which can harbour patent infections for years.^27^ C57BL/6 mice are rightly considered resistant because they effectively kill worms prior to the emergence of patent infection. The same strain dependence for primary infection largely extends to survival of the mF in the bloodstream, demonstrated by injection of naïve mice with mF taken from patent hosts. C57BL/6 mice eliminate mF rapidly from the bloodstream whereas BALB/c take months to fully clear transplanted mF.^25^, ^28^ This indicates that C57BL/6 mice are more resistant to a number of life cycle stages of *L sigmodontis*, from L4 to adult worms to mF.

## 5 ROLE OF LYMPHOCYTES

It is clear that mice require an adaptive immune system to control *L sigmodontis* infection and mF.^29^ Infected RAG2^−/−^IL2Rγ^−/−^ mice which lack T, B and NK cells, harbour more worms than wild‐type (WT) C57BL/6 controls and have microfilaraemia in the blood at day 72 p.i. which is never seen in WT C57BL/6 mice.^29^ T cells are required for worm killing in both resistant and susceptible mice. Depletion of CD4^+^ T cells from infected BALB/c mice increases worm burden and blood mF.^30^ We have completed similar experiments in C57BL/6 mice and found that depletion of CD4^+^ T cells resulted in a significant delay in worm killing (unpublished observation) (Tables 1 and 2).

**TABLE 1:** Outcome of reductionist studies in *L sigmodontis* infection using susceptible BALB/c mice and semi‐resistant strains (129/S and C3H)

Deficiency	Outcome	References
Lymphocytes (RAG2^−/−^IL4^−/−^)	↓ Worm killing, ↑ mF ↓ PC cells	[46]
B1 cells (‘Xid’ mice)	↓ Worm killing, ↑ mF, ↓ Th2 cytokines ↓ IgM ↓ IgG	[40]
B cells (‘B‐less’ mice)	= Worm killing, ↑ mF	[36]
B cells (‘μMT’ mice)	= Worm killing, ↓ mF, ↓ Worm development, ↓ Th1, ↓ and Th2	[31]
CD4^+^ T cells	↓ Worm killing, ↑ mF, ↑ IFN‐γ, ↓ Th2 cytokines ↓ IgE, ↓ EΦ	[30]
IFN‐γ	↓ Worm killing, ↓ Granulomas, ↓ NΦ, ↑ MΦ	[80]
IL4 or L‐4Rα	= Worm killing, ↑ mF, ↑ mF incidence, ↓ EΦ, ↑ NΦ, ↓ B‐cell proliferation, ↓ IgM, ↓ Lung inflammation	[36, 41, 44, 115]
IL‐5	↓ Worm killing, ↑ mF, ↓ EΦ, ↓ NΦ	[41, 44, 82]
IL‐4R and IL‐5	↓ Worm killing, ↑ mF, ↑ mF incidence ↑ Fibrosis, ↑ Th1 skewed	[43, 44]
IL‐5 + IFN‐γ	↓ Worm killing, ↓ NΦ, ↓ EΦ, ↓ Granulomas	[50]
IL‐12	= Worm killing	[80]
IL‐21	↓ mF, ↑ Th2 responses, ↓ Th1, ↑ IgM, ↑ IgG1.	[62]
IL‐10/IL‐10R	= Worm killing, ↑ BΦ IL‐4 production, ↑ IFN‐γ	[60, 75]
CD25/GITR/CTLA4	↑ Worm killing, ↓ Worm fitness	[60, 65]
PD1/PD‐L2	= worm killing, ↓ mF, ↑ Th2	[61]
NK cells	↓ Worm killing, ↓ mF killing/clearance	[73]
NKT cells	= Worm killing	[73]
Eosinophils	↓ Worm killing, ↑ mF, ↓ Pleural fibrosis, ↑ Worm development	[44, 46, 115]
EPO and MBP	↓ Worm killing (129/SvJ mice)	[83]
ST2	= Worm killing, ↑ mF (due to reduced splenic clearance), ↓ PC cells	[78]
IL‐33	= MΦ proliferation, ↓ M(IL‐4), worm readouts unknown	[120]
Basophils	= Worm killing	[74, 76]
CXCL12	= Worm killing	[117]
CCL17	↓ Worm killing	[79]
TLR4	↑ mF	[122]
TLR4	= mF	[123]
IL‐6	(Skin Phase) ↑ Early worm burden, ↑ EΦ, ↑ IgE, ↓ CCL17, ↓ NΦ, ↓ Mast cells	[128]
Eotaxin‐1	(Skin phase) ↓ Worm killing (in PC in late stage), = mF, ↓ EΦ IL‐6 production	[84]
Histamine‐R‐1	(Skin phase) ↓ larval establishment ↑ Worm killing (EΦ‐dependent), ↑ EΦ, ↓ IgE, ↓ IL‐5, ↓ IFN‐γ	[79, 85]
Mast cells	(Skin phase) ↑ larval establishment	[79]

Abbreviations: =, no change; ↑, increased; ↓, decreased; BΦ, basophil; EΦ, eosinophil; M(IL‐4), alternatively activated macrophages; MΦ, macrophage; NΦ, neutrophil; PC, .

**TABLE 2:** Outcome of reductionist studies in *L sigmodontis* infection using resistant C57BL/6 mice

Deficiency	Outcome	References
Lymphocytes (RAG2^−/−^ mice)	↓ MΦ proliferation, ↓ M(IL4) activation, worm readouts unknown	[98]
Lymphocytes (RAG2^−/−^IL2rg^−/−^mice)	↓ Worm killing, mice become mF^+^	[29]
B cells (Total, μMT mice)	= Worm Killing	[31, 34]
CD4^+^ T cells	↓ Worm killing ↓ MΦ proliferation ↓ M(IL‐4), ↓ EΦ	(UO)
IL‐4	↓ Worm killing, mice become mF^+^, ↑Th1	[34]
IL‐5	= Worm killing	[45, 46]
IL‐10/IL‐10R	= worm killing, ↑ T cell cytokines, ↑ Worm killing in IL‐4^‐/‐^ mice, ↑ clearance of mF challenge	[28, 67, 68, 69]
TGF‐β	↑ antigen‐specific T cell responses (via IL‐10), worm readouts unknown	[69]
IL‐17A	↓ Worms, ↑ Worm fitness, ↑ Th1‐skewed	[154]
TRIF	↓ Worm killing	[124]
NOD2	↓ Worm killing	[125]
CXCL12	↓ Worm Killing, ↓ PC cells	[117]
Granzyme A/B	↑ Worm killing, ↓ lymphocyte cell death, ↑ Th2 polarization, ↑ IgM	[71]
Neutrophils	= Worm killing, ↑ Worm killing in skin of mice with high basal skin NΦ (CXCR4^+/^ 13)	[129]

Abbreviations: =, no change; ↑, increased; ↓, decreased; M(IL‐4), alternatively activated macrophages; MΦ, macrophage; PC, peritoneal cavity; UO, unpublished observations.

Although B cells have a clear protective role in mediating vaccine‐induced immunity,^31^ the contribution of B cells to primary infection is less clear and may differ by strain. B cells are the most numerous cells of the pleural fluid and they expand greatly during *L sigmodontis* infection.^32^ This includes antibody‐producing B2 cells and innate‐like B1 cells.^33^ Parasite‐specific IgE and IgG1 are produced by both resistant and susceptible *L sigmodontis‐*infected mice.^34^ Although differences in B‐cell numbers between strains are not apparent in the pleural lavage, C57BL/6 mice exhibit sustained increases in all B‐cell subsets within the fat‐associated lymphoid clusters (FALCs) of the cavity.^33^ In contrast, BALB/c mice exhibit only a transient increase and fail to maintain B1b and B2 cells, which produce the majority of antigen‐specific IgM in the pleural cavity. The result is that BALB/c mice have far less *L sigmodontis*‐specific IgM than C57BL/6 mice by day 18 p.i.^33^ Release of IgM in the serous cavity by FALC B cells may represent a crucial source of protective antibodies, as serum IgM cannot diffuse into the body cavities. The potential importance of IgM for elimination of filarial larvae has been demonstrated in a closely related model of filarial infection, using sIgM^−/−^ mice.^35^

In an apparent contradiction, C57BL/6 ‘μMT’ mice which lack B cells are still able to kill worms and control mF.^34^ The same mutation backcrossed onto a BALB/c background showed no defect in late‐stage worm killing, but there were reduced mF counts and worm maturation, suggesting that B cells are somewhat protective. However, infected BALB/c μMT also exhibit reduced Th1 and Th2 response and macrophage accumulation.^31^ In contrast, ‘B‐less’, another B cell–deficient mouse on the BALB/c background, actually has increased mF (but no change in worm numbers) suggesting B cells play a role in control of patent infections.^36^ Total B‐cell deficiencies may paradoxically obscure the role of antibodies. Inconsistent results between different genetically altered B cell–deficient mouse strains maybe explained by the differential role of distinct B‐cell subsets. B cells, particularly B1 cells, have innate immune functions^37^ which include immune regulation.^38^ Thus, removing B cells from the C57BL/6 strain may alter worm killing even in the absence of host‐protective antibody. Supporting this hypothesis, BALB.Xid mice which have reduced B1 cells, particularly CD5^+^ B1a which are responsible for the production of antibodies to T‐independent antigens, harbour significantly more worms and higher blood mF than WT controls^39^, ^40^ (Tables 1 and 2).

### 5.1 T cells: key role for IL‐4

Immune responses to *L sigmodontis* are highly Th2 polarized in both resistant and susceptible strains but infected C57BL/6 mice exhibit dramatically higher local cytokine responses in the pleural cavity than BALB/c mice.^26^ IL‐4 is critical for resistance to *L sigmodontis* as IL‐4–deficient C57BL/6 mice resemble susceptible BALB/c mice, with equivalent adult parasite survival and circulating mF^34^ (Table 2).

The role of IL‐4 in susceptible BALB/c mice is not as straightforward. IL‐4–deficient or IL‐4Rα–deficient BALB/c mice, harbour similar numbers of worms as WT BALB/c mice late in infection indicating that IL‐4 is not responsible for killing of adult worms in this strain.^36^, ^41^, ^42^ However, IL‐4 greatly limits the mF counts in BALB/c mice.^36^, ^41^, ^42^ Infected IL‐4–deficient BALB/c mice also have reduced eosinophils but increased neutrophils.^36^ In contrast to IL‐4 deficiency, IL‐5 deficiency on the BALB/c background does increase adult survival,^41^ and a dramatic effect is seen in IL‐4Rα^−/−^/IL‐5 mice which harbour more worms and at least a hundred‐fold increase in blood mF.^43^, ^44^ In contrast, C57BL/6 IL‐5–deficient mice are equally resistant to WT controls,^45^, ^46^ highlighting another significant strain difference, as discussed below with regard to eosinophils. It is important to note that IL‐13 has not been specifically investigated in *L sigmodontis* infection and that IL‐4–deficient mice on the C57BL/6 background have a profound IL‐13 defect while IL‐4Rα–deficient mice do not distinguish the contribution of IL‐4 and IL‐13 (Table 1). Given that GWAS studies of onchocerciasis show association of IL‐13 polymorphisms with the development of pathology,^47^, ^48^ an investigation of the role of IL‐13 is warranted.

Patients with onchocerciasis and lymphatic filariasis typically present with a Th2‐polorized phenotype, but many patients have a more mixed Th1/Th2 T‐cell phenotype, especially those with pathology.^9^, ^10^, ^49^ IFN‐γ and Th1 cells increase along with Th2 cytokines in both resistant and susceptible strains of mice infected with *L sigmodontis.* ^26^, ^50^ Interestingly, transcripts for IFN‐γ peak when mF are first produced in BALB/c mice.^51^ IFN‐γ and IL‐5 synergize to enhance worm killing in late‐stage infection of BALB/c mice, indicating a cooperation of Th1 and Th2 cells leading to worm killing.^50^

### 5.2 T‐cell regulation and Hyporesponsiveness

Immune regulation and immune hyporesponsiveness are features of chronic helminth infection including filariasis.^52^, ^53^, ^54^, ^55^ The most common outcome in human filariasis is mild/asymptomatic disease despite the presence of adult worms and mF, reflecting a remarkable degree of tolerance by the host.^1^, ^6^, ^8^, ^9^ This can be explained by active immunoregulation on part of the parasite but also host regulatory networks, which act in concert to prevent immunopathology.^56^, ^57^, ^58^ Pathological symptoms in onchocerciasis and lymphatic filariasis are often associated with immune responses to dead/dying worms which remain in the tissue, indicating that tolerance to established parasites can be more beneficial to the host than ‘protective’ worm killing.^1^, ^6^, ^8^, ^9^, ^10^

**FIGURE 4:**
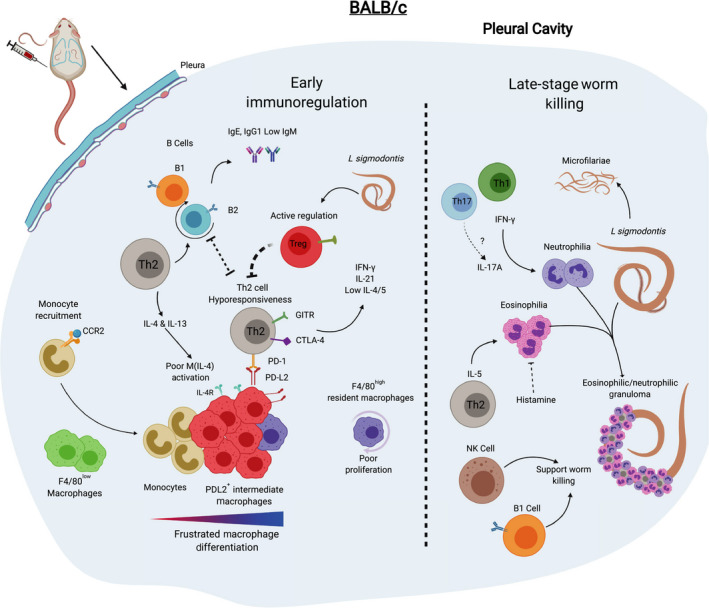
Immune response to *L sigmodontis* infection in BALB/c is characterized by immunoregulation and delayed worm killing. In infected BALB/c mice, there is a relatively weaker accumulation of immune cells in the pleural cavity in comparison with C57BL/6 mice. The immune response is initially Th2‐biased with associated eosinophilia and B‐cell production of IgG1 and IgE. The T‐cell response shifts towards a hyporesponsive state by day 40 p.i. Th2 cells express GITR, CTLA‐4 and PD‐1 which facilitate worm survival. Regulatory T cells also limit T‐cell responses and worm killing. In BALB/c mice, there is comparatively less F4/80^high^ macrophage proliferation than in C57BL/6 mice and these are instead outnumbered by incoming CCR2‐dependent monocytes which develop into PD‐L2^+^ macrophages with a phenotype that is intermediate between F4/80^low^ and F4/80^high^ macrophages. Sexually mature *L sigmodontis* female worms produce mF beginning around day 55 p.i., and late‐stage (day 80 p.i. onwards) worm killing occurs via the gradual encasement of worms in granulomas. B1 and NK cells play a supporting role in worm killing. IFN‐γ supports neutrophilia and granuloma formation. IL‐5 and eosinophils are required for worm killing in BALB/c mice

Filarial nematode infection of humans is associated with regulatory and hyporesponsive Th1 and Th2 responses, especially in heavily infected individuals without pathology.^49^, ^54^, ^58^, ^59^ GWAS studies have linked polymorphisms in CTLA4 and TGFB1, genes linked with T‐cell regulation, to susceptibility to filariasis.^48^ *L sigmodontis* has proved a powerful model system to study regulation of Th2 responses. Th2 cells in BALB/c mice infected with *L sigmodontis* acquire features of hyporesponsiveness that are consistent with human studies.^51^, ^60^, ^61^, ^62^ In BALB/c mice, Th2 cytokine production is relatively high in early‐stage infection (up to day 40). However, by the mid‐stage infection (days 40‐60), prior to the appearance of mF, Th2 cytokine production is notably reduced.^51^, ^60^, ^61^, ^62^ Following the production of mF however, there is a recovery in IFN‐γ and Th2 cytokines which coincide with worm killing in BALB/c mice (Figure 4).^51^

Mechanistically, T‐cell hyporesponsiveness can be viewed as being cell‐intrinsic (hyporesponsiveness of Th2 cells themselves) or cell‐extrinsic (active regulation by other factors). A recent study has provided evidence that T‐cell hyporesponsiveness is intrinsic to the Th2 cell.^62^ By day 60 p.i., Th2 cells in infected BALB/c mice cells acquire a dysfunctional phenotype that is IL‐21^+^Egr2^+^c‐Maf^+^Blimp‐1^+^IL‐4^lo^IL‐5^lo^T‐bet^+^IFN‐γ^+^. Critically, this phenotype is maintained upon transfer of these cells to naïve mice.^62^ An intriguing feature of these cells is the production of IL‐21, which appears to contribute to mF survival while limiting germinal centre B cells and parasite‐specific IgM and IgG1.^62^

There is also evidence of extrinsic regulation of T cells in *L sigmodontis*‐infected BALB/c mice. Th2 cells acquire PD1 expression, and signalling from PD‐L2 reduces Th2 cytokine production, and increases blood mF and worm fecundity.^61^ Monocyte‐derived pleural cavity macrophages are the likely source of PD‐L2.^32^ In addition, pleural cavity F4/80^+^ macrophages (but not DC or B cells) suppress T cells, at least partially, via TGF‐β.^63^ Likewise, Treg cells play a key role in susceptibility of BALB/c mice to infection with *L sigmodontis.* CD25^+^ Treg cells expand in infected BALB/c mice.^60^, ^64^, ^65^ Moreover, Foxp3^+^ CD4^+^ T cells enhance their expression of CD25, Foxp3, GITR, ICOS and CTLA‐4.^64^, ^65^ Anti‐CD25 and anti‐GITR treatment leads to an increase in Th2 cytokines and a significant increase in worm killing.^60^ In vivo neutralization of CTLA‐4 and depletion of CD25^+^ cells also enhances worm killing.^64^ Furthermore, a single depletion of CD25^+^ cells prior to infection increases worm killing, decreases worm fecundity and reduces blood mF in late‐stage infection (Figure 4, Table 1).^65^ Collectively, these results reveal that T‐cell hyporesponsiveness and regulation plays a critical role in susceptibility to *L sigmodontis* infection in BALB/c mice (Figure 4).

C57BL/6 mice clear the infection before the onset of the hyporesponsive phenotype seen in BALB/c mice. Th2 cytokine concentrations are higher in the infected tissue of C57BL/6 than BALB/c mice indicating a more vigorous effector response in those animals.^26^ Although less well studied than BALB/c mice in this context, C57BL/6 mice do acquire some immunoregulatory features. Treg cells expand in infected C57BL/6 mice,^66^ although they proliferate less than Treg cells in BALB/c mice.^65^ IL‐10–deficent C57BL/6 mice display exaggerated worm specific Th1 and Th2 responses.^67^ While IL‐10–deficient animals do not harbour more worms,^67^ the failure to kill worms in IL‐4–deficient C57BL/6 mice is reversed if those mice also lack IL‐10.^68^ Moreover, TGF‐β–induced IL‐10 production by T cells in *L sigmodontis‐*infected C57BL/6 mice mediates bystander suppression of vaccine‐induced immune responses.^69^ Granzyme A/B expression is a feature of activation‐induced autologous T cells associated with immunosuppressive responses to dying worms in onchocerciasis.^70^ In C57BL/6 mice, granzymes A and B may promote early worm survival, suppressing type 2 immune responses and limiting parasite‐specific IgM.^71^

Together, these data provide evidence that the susceptibility phenotype in BALB/c mice relies on a robust T regulatory and hyporesponsive Th2 response that is less evident in C57BL/6 mice. This divergence in effector Th2 vs regulated Th2 responses positions *L sigmodontis* as a model system to study the genetic basis of the control of Th2 cell fate.

### 5.3 ILC, NK cells, basophils and mast cells

The numbers of NK cells, basophils and type 2 innate lymphoid cells (ILC2s) increase over the course of infection in BALB/c mice,^72^, ^73^, ^74^, ^75^, ^76^ but little is known about their function. These cells are present when CD4^+^ T cells are depleted, which enhances worm survival, and thus they are not sufficient to kill the parasite. However, they might play a supporting role in the establishment or maintenance of protective Th2 responses. Depletion of NK cells delays late‐stage worm killing and enhances mF survival,^73^ but depletion of basophils has no effect on worm killing or mF counts*.* ^74^, ^76^ Basophil depletion does, however, reduce parasite‐specific IgE, circulating eosinophils and IL‐4 production by T cells.^74^ ILC2s and basophils are targets of IL‐33.^77^ Loss of the IL‐33 receptor (ST2) in BALB/c mice does not affect worm killing although these mice do harbour more mF due to a defect in splenic clearance of blood mF.^78^ *L sigmodontis*‐infected ST2‐deficient mice have normal Th2 responses but reduced cell recruitment to the site of infection.^78^ Another target of IL‐33, mast cells, are increased following infection of semi‐susceptible C3H/HeN Mice. Moreover, mice deficient in CCL17, a chemotactic factor for mast cells had a fourfold increase in parasite burden.^79^ That study suggests that mast cell degranulation, which enhances vascular permeability, may facilitate larval migration.^79^ Mast cells may thus play a supporting role in establishment of early *L sigmodontis* infection, but we do not know whether they affect the outcome of infection in the pleural cavity (Table 1). The absence of studies for these innate cells, or the IL‐33‐ST2 axis, in C57BL/6 mice means we are currently ignorant of their contribution to innate resistance.

## 6 WORM KILLING, GRANULOMAS, EOSINOPHILS AND NEUTROPHILS

We do not yet have a detailed understanding of the mechanisms by which *L sigmodontis* parasites are killed but it does involve the gradual encasement of the worm within a granuloma. Cuticles that are shed during moulting are rapidly encased in granulomas formed of eosinophils.^27^ However, the living worm represents a more difficult target due to its high motility and selective pressure to avoid exposing residues that enable immune cell attachment.^27^

In the fully permissive Mongolian jird, granulomas contain neutrophils as well as eosinophils.^27^ In BALB/c mice granuloma formation around young adult worms is rare but develops later in infection around larger adult worms and contains neutrophils which form the immediate layer of the granuloma exposed to the worm.^27^ In BALB/c mice neutrophils are recruited relatively late in infection prior to the onset of worm killing.^50^, ^80^ Late‐stage granuloma formation and worm killing is attenuated in IFN‐γ–deficient animals which have a deficiency in neutrophil recruitment, but exhibit no difference in mF counts.^80^ Neutrophils from infected IFN‐γ–deficient mice have reduced chemotactic and phagocytotic ability ex vivo and pleural cavity cells produce less TNF‐α and NO in response to M1 stimuli.^80^ These data are strongly suggestive of a role for neutrophils in the eventual killing of the adult parasites in BALB/c mice. Notably, neutrophils are not a significant feature of *L sigmodontis* infection in resistant C57BL/6 mice (Table 1).

In both C57BL/6 and BALB/c mice, eosinophils increase in the pleural cavity as early as 11 days p.i.,^32^ and recruitment is much reduced in mice lacking CD4^+^ T cells.^29^, ^30^ Hyper‐eosinophilic IL‐5‐transgenic mice on the semi‐susceptible CBA background are more resistant to infection^81^ supporting a role for eosinophils in filarial killing. Further evidence comes from primary infection of IL‐5–deficent BALB/c mice, which have much greater late‐stage worm numbers and higher blood mF.^41^ In fact IL‐5 has a greater effect on worm/mF numbers than the absence of IL‐4.^41^ Infected BALB/c mice also harbour more late‐stage worms and mF following administration of an anti‐IL‐5 antibody.^82^ In addition to low eosinophil numbers, IL‐5–deficient mice have significantly less B‐cell accumulation and IgM production at the site of infection,^41^ which might further support a role for IgM as an anti‐worm effector. A direct role for eosinophils is supported by infection of semi‐resistant 129/SvJ mice that lack key eosinophil granule proteins major basic protein (MBP) or eosinophil peroxidase (EPO). These mice harbour more worms than even susceptible BALB/c mice at day 28 p.i. implicating eosinophil degranulation as an important factor in worm killing in BALB/c mice.^83^ Notably, BALB/c mice deficient in Eotaxin‐1 have increased late‐stage worm counts without any change of mF counts.^84^ Further evidence comes from the finding that IL‐4Rα^−/−^, IL‐5^−/−^, dblGATA (eosinophil deficient) and IL‐4R^−/−^/IL‐5^−/−^ mice on BALB/c background all display increased worm burdens and mF in the blood in late‐stage *L sigmodontis* infection.^44^ Of the four strains assayed, IL‐4R^−/−^/IL‐5^−/−^ display the greatest increase in susceptibility (Tables 1 and 2).^44^ Finally, administration of anti‐histamine to infected BALB/c enhances clearance of adult worms in an eosinophil‐dependent manner, indicating that parasite‐induced histamine impairs anti‐parasite immune responses by limiting eosinophil activation.^85^

Granuloma formation is less apparent in BALB/c mice lacking both IFN‐γ and IL‐5^50^ and neutrophil recruitment and activity is markedly decreased in these mice, with greater worm burdens than mice deficient in either cytokine alone.^50^ GM‐CSF a key survival and proliferative factor for both eosinophils and neutrophils has a greater inhibitory effect on worm killing than IL‐5.^82^ Interestingly, neutralization of IL‐5 in infected BALB/c mice, which reduces worm killing, is associated with reduced neutrophil accumulation,^82^ indicating an unexpected cooperation between neutrophils and eosinophils in late‐stage worm killing in BALB/c mice (Figure 4, Table 1).

**FIGURE 5:**
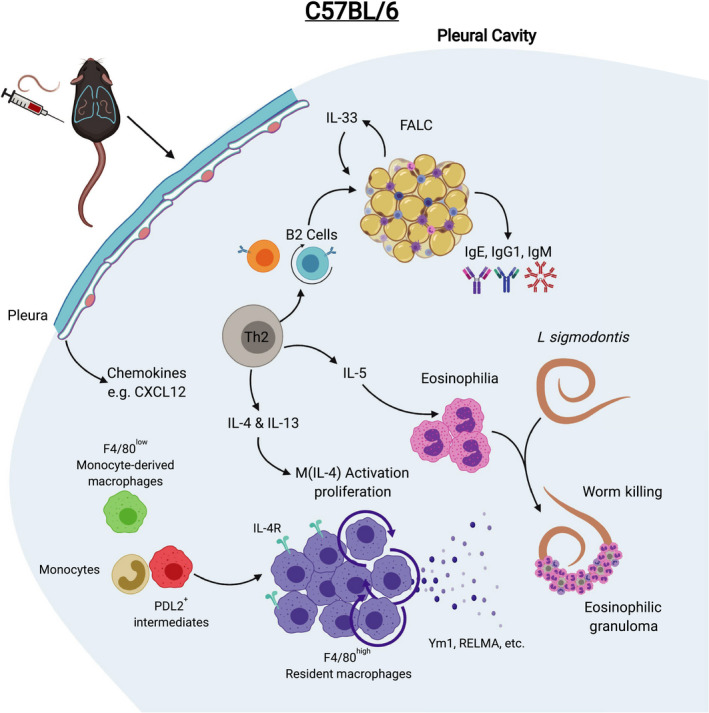
Immune response to *L sigmodontis* infection in C57BL/6 mice leads early to worm killing in the pleural cavity. C57BL/6 mice mount an early and sustained Th2‐biased immune response to *L sigmodontis* characterized by greater cell accumulation in the pleural cavity than in BALB/c mice. This is associated with eosinophilia, proliferation of B2 cells and the development of FALCs within the pleural and pericardial cavities and the production of parasite‐specific IgE, IgG1 and IgM. The pleura supports resistance by the production of chemokines including CXCL12. F4/80^high^ macrophages proliferate and greatly expand in number and become M(IL‐4) activated producing Ym1 and RELM‐α. Monocytes, F480^low^ macrophages and PD‐L2^+^ intermediate macrophages are present but outnumbered by F4/80^high^ macrophages. Dead worms encased in eosinophilic granulomas are detectable by day 35 p.i., and infection is rapidly cleared thereafter

Evidence for eosinophils mediating *L sigmodontis* worm killing in resistant C57BL/6 is less clear. In C57BL/6 mice, granulomas form along the lateral lines of small newly moulted adult worms.^27^ Granulomas in these mice are predominantly eosinophilic (unpublished observation). However, there is no deficit in worm killing following primary infection of IL‐5–deficient C57BL/6 mice (Figure 5).^45^, ^46^ This finding, that infected IL‐5–deficent C57BL/6 mice, which cannot attract eosinophils have no defect in worm killing is hard to square with the idea that granulomas are required to kill worms (Table 2).^45^ This raises the possibility that granulomas in C57BL/6 may form around already dead worms, like they do around shed cuticles rather than being a key component in worm killing itself.

A far more straightforward role for eosinophils exists in killing of larval parasites during secondary infection. In both C57BL/6 and BALB/c mice, eosinophils are rapidly recruited to the skin and degranulate where they mediate larval killing; IL‐5–deficent mice of both strains lose protection following vaccination.^45^, ^86^ This is consistent with a greater role for eosinophils in larval killing over adult worm killing seen for other helminths.^87^ The evolutionary importance of eosinophils in parasite control is perhaps best illustrated by the finding that, in both BALB/c and C57BL/6 mice, *L sigmodontis* L3 larvae respond to the presence of eosinophils by accelerating their development.^46^ Thus, the parasite appears able to detect cues that a host is mounting an effective immune response.

Taken together, these results show that killing of adult *L sigmodontis* is highly strain dependent. Both eosinophils and neutrophils contribute to worm killing and mF control in late‐stage infection in BALB/c mice but killing of immature worms in resistant C57BL/6 is still poorly defined.

## 7 MACROPHAGES OF THE PLEURAL CAVITY

There are two distinct macrophage populations in the pleural cavity, F4/80^high^ and F4/80^low^ macrophages which are similar to macrophages of the other serous cavities, the pericardial and peritoneal cavities (Figure 1). The F4/80^high^ and F4/80^low^ macrophages correspond to the large peritoneal/pleural macrophages (LPM) and small peritoneal/pleural macrophages (SPM) respectively, well described for the peritoneal cavity^88^ but more recently described in the pleural cavity.^32^, ^89^, ^90^, ^91^ However, peritoneal macrophages remain better studied and much of the basic biology of the pleural cavity cells must be extrapolated from peritoneal studies.

Serous cavity F4/80^low^ macrophages are monocyte‐derived and develop only after birth. There is some heterogeneity within the population which might represent stages of development into a terminally differentiated CD11c^+^ CD226^+^ cell.^91^ F4/80^low^ macrophages have a half‐life of about 2 weeks in vivo being replaced by CCR2^+^ monocytes but there is a subset that are CD115^−^CD11c^+^ and not replaced via CCR2‐mediated recruitment, with overlapping features of DC.^92^ The major phenotypic feature which discriminates F4/80^low^ from F4/80^high^ macrophages is their high expression of MHC‐II. F4/80^low^ macrophages also express more genes involved in antigen presentation and are capable APCs, a feature lacking in F4/80^high^ macrophages.^93^, ^94^ F4/80^high^ resident macrophages are seeded to the serous cavities before birth from foetal hematopoietic stem cells.^95^, ^96^, ^97^ F4/80^high^ macrophages have the capacity to self‐renew by proliferation in the serous cavities, which relies on CSF1.^92^, ^98^ CSF1R–deficient mice have reduced basal macrophage proliferation and lower F4/80^high^ macrophage numbers.^98^ However, it is now clear that F4/80^high^ macrophages are eventually replaced by cells of bone‐marrow origin.^92^, ^96^, ^99^ Replacement is gradual and requires incoming monocytes.^100^ In CCR2^−/−^ mice, which lack monocyte‐derived cells, there is a loss in the number of F4/80^high^ macrophages suggesting that in naïve mice basal proliferation of embryonically derived F4/80^high^ macrophages is insufficient to maintain the population.^92^ Monocytes that develop into F4/80^high^ macrophages do so via a cell which resembles an F4/80^low^ macrophage but with intermediate phenotypic features of an F4/80^high^ macrophage.^92^, ^101^ Newly differentiated F4/80^high^ macrophages largely phenocopy embryonically derived F4/80^high^ macrophages save for a failure in some cells to express Tim4, a marker of residency in other tissues.^92^, ^102^

F4/80^high^ macrophages survive in the tissue for much longer than F4/80^low^ macrophages with a half‐life of 12 weeks for peritoneal macrophages.^89^, ^92^ GATA6^+^ is the key transcription factor for control of the serous cavity ‘residency’ transcriptional programme.^103^, ^104^, ^105^ Loss of GATA6 from myeloid cells impairs the development and proliferative capacity of F4/80^high^ macrophages. The macrophages that do develop in GATA6^−/−^ mice have a partially differentiated phenotype, resembling macrophages that are differentiating into F4/80^high^ macrophages from monocytes in naïve mice with a failure to express ‘residency’ genes.^103^, ^104^ F4/80^high^ macrophage development also requires C/EBP‐β and a similar partially differentiated phenotype is observed in C/EBPβ‐deficient animals.^16^

### 7.1 M(IL‐4) activation and proliferation in *L sigmodontis infection*

Leaving aside lymphocytes, the most abundant cells in the pleural cavity of infected C57BL/6 mice are macrophages and eosinophils. The fact that IL‐5 was found to be dispensable for worm killing in C57BL/6 mice while IL‐4 is required highlights the potential importance of macrophages in *L sigmodontis* infection due the capacity of IL‐4Rα signalling to potently activate macrophages to become M(IL‐4) cells.^17^

*L sigmodontis* infection induces M(IL‐4) cells in both C57BL/6 and BALB/c mice.^29^, ^98^, ^108^, ^109^ In late‐stage BALB/c infection macrophages have a marked M(IL‐4) phenotype with high expression of arginase‐1, Ym1 and RELM‐α.^19^ However, our unpublished data have revealed that F4/80^high^ macrophages from BALB/c mice, on a cell‐to‐cell basis, are much less M(IL‐4)‐polarized than in C57BL/6 mice. They have similar expression of RELM‐α and arginase‐1 but lack many of the genes that are characteristic of M(IL‐4) activation in F4/80^high^ macrophages.^110^ The relative lack of M(IL‐4) polarization may be a factor in the susceptibility of BALB/c mice to *L sigmodontis* infection.

In the pleural cavity of *L sigmodontis‐*infected C57BL/6 mice, F4/80^high^ macrophages greatly expand in number, beginning early in infection and continuing throughout infection, eventually becoming the most abundant cell type.^32^, ^111^ This is accompanied by only limited recruitment of monocytes and relative loss in the proportion of F4/80^low^ macrophages*.* ^111^ The pleural cavity F4/80^high^ macrophages undergo a burst of proliferation peaking at 10 days p.i. which is dependent on IL‐4 and occurs without the input of blood monocytes (Figure 5)*.* ^111^ This finding was the first demonstration that macrophages could expand dramatically in number through local proliferation rather than blood cell recruitment during an inflammatory response. The paradigm shift in macrophage biology that resulted was a direct consequence of the *L sigmodontis* model. Macrophages that can respond to IL‐4 outcompete, through proliferation, macrophages which lack IL‐4Rα^98^ and proliferation is independent of the CSF1R highlighting the direct role of IL‐4 in macrophage proliferation.^98^ The cellular source of IL‐4 is not known but given that macrophages fail to proliferate in *L sigmodontis‐*infected RAG‐deficient mice, an adaptive immune cell is involved.^98^ Our unpublished data indicate that the proliferation and expansion of F4/80^high^ macrophages is dependent on the recruitment and activation of CD4^+^ T cells. This suggests that innate sources of IL‐4, such as basophils, eosinophils and ILC2 are unlikely to be capable of inducing the proliferation of macrophages during infection.

The situation in BALB/c mice is markedly different. F4/80^high^ macrophage proliferation is relatively weaker in *L sigmodontis‐*infected BALB/c mice. There is an accumulation of monocytes and monocyte‐derived F4/80^low^ macrophages These macrophages are highly heterogenous and appear to contain partially differentiated macrophages. This results in a much smaller accumulation of F4/80^high^ macrophages at the site of infection in BALB/c mice when compared to C57BL/6 mice (Figure 4).^32^

F4/80^high^ and F4/80^low^ serous cavity macrophages respond differently to IL‐4Rα signalling.^112^ For example, F4/80^high^ macrophages uniquely upregulate *Ucp‐1* and cell cycle genes while F4/80^low^ macrophages have greater PD‐L2 expression, retinoic acid production and the unique ability to induce the differentiation of Foxp3^+^ Treg cells.^112^ These unique responses to IL‐4 suggest that F4/80^high^ and F4/80^low^ macrophages will play different roles in *L sigmodontis* infection. Indeed, macrophages from BALB/c mice are immunoregulatory and promote worm survival. Infection of BALB/c mice leads to the recruitment of monocyte‐derived macrophages which express PD‐L2 (Figure 4).^32^ Blockade of monocyte recruitment in infected BALB/c mice increases T‐cell IL‐4 production with enhanced worm killing.^32^ This is highly consistent with previous data showing that antagonism of the PD‐L2/PD1 pathway reverses Th2 cell exhaustion and decreases worm fitness and mF counts in late‐stage *L sigmodontis* infection of BALB/c mice.^61^ Thus, it appears that monocyte‐derived macrophages in BALB/c mice promote worm survival by limiting Th2 cell activity. F4/80^high^ resident macrophages from BALB/c mice, although far fewer in number, may also be immunoregulatory as arginase‐1^+^F4/80^high^ resident macrophages taken from infected BALB/c mice potently inhibit the proliferation of T cells in vitro, partially via TGF‐β.^63^

Efforts to address the contribution of F4/80^high^ resident M(IL‐4) macrophages to worm killing have been hampered by difficulties in effectively deleting IL‐4Rα due to lack of penetrance using Cre recombinase technology. In the strong IL‐4 environment of *L sigmodontis* infection, the few cells that still express the receptor outcompete the IL‐4Rα–deficient cells by local proliferation.^113^ However, in a study of BALB/c mice using LysMCre^+^ X IL‐4Rα^fl/fl^, mF numbers were significantly higher than controls, despite the fact that the macrophages had reverted to a WT phenotype by the time the infection became patent.^113^ This suggests that early in infection M(IL‐4) macrophages contribute to a pathway leading to containment of mF later in infection.

## 8 THE PLEURAL NICHE

Given the presence of inflammatory infiltrate, oedema and motile worms, it is not surprising that the stroma of the tissue niche undergoes transformations during *L sigmodontis* infection.^17^, ^43^, ^114^, ^115^ Progressive fibrotic pathologies develop in the pleura of BALB/c mice infected with *L sigmodontis* ^43^, ^115^ along with inflammatory foci in the lungs.^17^, ^43^, ^114^

The effect of the pleura itself upon pleural immune cells and the outcome of infection is largely unknown. We have previously shown that tissue‐specific niche‐derived factors support IL‐4Rα–dependent proliferation of tissue‐resident macrophages.^116^ C1q, a complement component, acts to enhance F4/80^high^ peritoneal macrophage proliferation and activation in the peritoneal cavity in response to IL‐4, while surfactant protein A performs the same function in the lung. Both factors act through the atypical myosin, Myo18A However, pleural cavity F4/80^high^ macrophages did not express Myo18A.^116^ Nevertheless, the concept that the niche can provide factors which support macrophage proliferation and M(IL‐4) activation is relevant to *L sigmodontis* infection.

The pleura and in particular the mesothelium play an active role in the maintenance of macrophages in the serous cavities. For example, retinoic acid (RA) produced by WT1^+^ mesothelial cells maintains GATA6 expression in F4/80^high^ macrophages, which is required for its ‘residency’ programme.^89^ RA and IL‐4 mediate the conversion of thioglycolate‐elicited F4/80^low^ macrophages into F4/80^high^ macrophages.^11^ In the absence of RA, macrophages do not fully convert into F4/80^high^ macrophages remaining in an intermediate PD‐L2^+^ phenotype.^11^ This phenotype resembles the phenotype of PD‐L2^+^ F4/80^low^ macrophages that we find in BALB/c mice infected with *L sigmodontis*.^32^ This suggests that RA signalling deficiencies may contribute to the accumulation of this immunoregulatory cell in infected BALB/c mice.

In evidence for the role of the tissue niche in *L sigmodontis* infection, mesothelium was shown to be critical for the recruitment of cells to the pleural cavity. CXCL12 is produced by the mesothelium in infected‐C57BL/6 but not BALB/c mice.^117^ Chemical blockade of the CXCL12‐CXCR4 pathway significantly reduced immune cell recruitment and reduced worm killing in C57BL/6 mice but had no effect in BALB/c mice.^117^ Another evident difference between strains is the expansion of the FALCs, which is significantly greater in infected C57BL/6 mice in comparison to BALB/c mice.^33^ IL‐33 is produced by FALC stroma,^33^ and thus, IL‐33 may be more abundant in C57BL/6 mice during *L sigmodontis* infection. IL‐33, which is also expressed in the lining of the lung and by mesothelial cells in other organs,^77^, ^118^ is a diagnostic marker of pleural effusion.^119^ Our unpublished data indicate that macrophages from C57BL/6 but not BALB/c mice upregulate ST2 during infection. Consistent with this finding and the potentially lower availability of IL‐33 in BALB/c mice,^33^ ST2 or IL‐33‐deficency in BALB/c mice does not affect worm killing in BALB/c mice, although ST2‐deficient mice have less recruitment of eosinophils, CD4^+^ T cells and macrophages to the site of infection.^78^, ^120^ In BALB/c mice IL‐33 is not required for macrophage proliferation with *L sigmodontis* infection.^120^ However, BALB/c mice may not have been a relevant strain to investigate macrophage proliferation as they display reduced macrophage proliferation in comparison to C57BL/6.^32^ Given its potential to produce cytokines, chemokines or other factors such as RA which support F4/80^high^ macrophages, the pleura certainly has the potential to impact upon the outcome of infection. Fundamentally, we do yet not know whether resistance to infection seen in different strains of mice is mediated by immune cells or if the tissue niche dictates the outcome.

### 8.1 Beyond the pleural cavity

This review has focused on the local immune responses to *L sigmodontis* in the pleural cavity. Thus, we have neglected to mention responses which occur early in infection in the skin and evidence for *L sigmodontis* having effects on immune responses in distal sites.

Most *L sigmodontis* L3 larvae do not survive their journey from the skin and relatively little is known about the responses in the skin apart from the aforementioned contribution of mast cells in primary infection^79^ and eosinophils in secondary infection.^9^, ^45^, ^86^ *L sigmodontis* contain *Wolbachia*, bacterial endosymbionts which contain ligands that may activate innate sensing pathways in the skin,^29^ and there is evidence that TLR2,^121^ TLR4,^122^, ^123^ TRIF^124^ and NOD2^125^ all contribute to the immune response to *L sigmodontis* infection. Innate lymphoid cells and γδ T cells are major players in skin barrier function during type 2 immunity,^126^, ^127^ but have not been investigated in the context of infection by filarial larvae (Tables 1 and 2). Combined data from two papers suggest that mast cells via IL‐6 activation and CCL17 recruitment may contribute to killing of *L sigmodontis* larvae in the skin following primary infection.^79^, ^128^ In addition, neutrophils have the capacity to kill L3 larvae in the skin and are recruited to the skin following infection.^125^, ^129^ However, neutrophil depletion in WT C57BL/6 mice had no effect on adult worm numbers,^129^ leaving open the contribution of neutrophils to early resistance. The contribution of the skin to innate resistance, and perhaps more importantly, the impact the immune response to the migrating larvae in the skin/lymphatics has upon the later protective immune responses in the different strains remains an unresolved question.

In keeping with the extensively studied immune regulatory/suppressive effects of helminth infection,^53^ *L sigmodontis* infection or products protects mice against *E‐coli*–induced sepsis,^121^ diet‐induced glucose intolerance,^130^ type‐1 diabetes*,* ^131^, ^132^ atherosclerosis^18^ and allergic responses.^133^, ^134^ These studies provide insights into new helminth‐mediated regulatory pathways such as the suppression T‐cell response by interference with the development of cytotoxic CD8^+^ T cells,^135^ follicular helper T cells,^136^ or by TGF‐β.^69^, ^132^ These results highlight the capacity of *L sigmodontis* to induce global immune alterations in the host best exemplified by the parasites ability to interfere with vaccine‐induced protective immunity against, *Plasmodium*,^135^ Friend virus^137^ and influenza.^66^ Fitting these findings, and perhaps partly explaining them, is the remarkable finding that peritoneal implantation of a single adult female (but not male) delays the elimination of exogenously delivered blood mF, suggesting that the parasite acts systemically to protect her offspring.^28^

## 9 FUTURE PERSPECTIVES

We believe that the unique features of the *L sigmodontis* model will reveal further insights into myeloid cell function, as well cellular communication across tissues and between immune and stromal cells. Results from our laboratory strongly suggest a key role for pleural cavity macrophages in *L sigmodontis* infection but the heterogeneity of these cells has yet to be described accurately. In both resistant and susceptible mice, we observe cells which fall under the banner of M(IL‐4) cells but in fact these are an amalgam of cells of diverse origins and activation states. New technologies such single‐cell sequencing, mass spectrometry and high‐parameter cytometry will allow us to better describe complex myeloid populations without the bias of ‘gates’. The pleural cavity is a relatively simple organ system which also represents a model to study niche‐immune cell interactions such as ligand‐receptor analysis that will provide a more holistic understanding of immune cell function and interactions. More importantly, we have yet to convincingly link these macrophages to worm killing. The use of novel genetically modified mice may help to answer these questions. In particular, Ms4a3‐reporter mice will allow for fate mapping of monocyte‐derived cells,^10^ macrophage‐specific GATA6‐deficent mice will allow us to ascertain the role of the residency programme in F4/80^high^ cells in resistance,^15^ and mice with a cell‐specific deletion in Bhlhe40 can be used to ascertain the importance of F4/80^high^ macrophage proliferation^138^ to infection outcome.

The variation in susceptibility across many mouse strains (Figure 2) paints *L sigmodontis* infection as a suitable candidate for mapping susceptibility to genetic loci using haplotype lineage analysis or quantitative trait locus. The fact that only mice of the BALB lineage develop mF after infection limits susceptibility loci to that mouse. Many common mouse strains are historically derived from 19th‐century mouse fanciers who bred mice for behavioural and aesthetic traits. Thus, rather than being truly separate, inbred mouse lines share much common ancestry and are predominantly derived from the *Mus musculus domesticus* subspecies.^139^, ^140^, ^141^ The most common strains of mice only represent a small portion of the diversity in alleles amongst wild mice.^142^ The resistance of relatively new wild‐derived strains to *L sigmodontis* is of considerable interest, as this may provide more accurate determination of genetic loci, without the need for genotyping.^139^, ^143^ This would be achieved by the use of recombinantly inbred mouse lines which make use of wild‐derived mice, such as the Collaborative Cross, that have been created to minimize the number of strains required to capture maximal genetic diversity.^142^

There are now thought to be in the region of 450 mouse strains.^144^ However, if anything the number of strains in common use has been in steep decline for decades. One reason is the wide availability of genetically altered mice only on certain backgrounds and the fact that the C57BL/6J mouse is the reference genome.^145^ This gradual acceptance of the dominant position of C57BL/6 mice raises the issue that many basic discoveries in immunology may be mouse strain‐specific. Indeed, it was the *L sigmodontis* model in C57BL/6 mice that allowed the paradigm‐shifting discovery that macrophages could increase dramatically in number through local proliferation, without requirement for blood‐derived cells during an inflammatory response.^111^ Similar experiments in BALB/c mice would not have revealed the same myeloid cell dynamics. Nonetheless, the basic biology holds true in other strains but to widely varying extent^32^ and the comparison itself is now proving powerful. It is thus imperative to be aware that recent breakthroughs in myeloid biology have largely been achieved using only C57BL/6 mice.^95^, ^96^, ^97^, ^138^ Since the first extension of the Th1‐Th2 dichotomy to macrophages,^146^ the genetic underpinnings of these phenotypes have remained largely elusive. Despite their hybridized ancestry, inbred strains of mice harbour an astonishing amount of loss of function mutations*.* ^147^, ^148^, ^149^ Ultimately, cell identity and function in a type 2 immune environment will depend less on simple allelic differences and more upon transcription factor interactions.^150^, ^151^ The biological consequences provided by differences in cis‐regulatory elements in different strains of mice have already been shown to have profound effects on macrophage transcription factor interactions and cell function.^152^, ^153^ We are unlikely to be able to model the functional consequences of such genomic differences any time soon. Until such time, we will need to pay attention to the repertoire of genetic diversity provided by extant inbred mouse strains.

## CONFLICT OF INTEREST

None.
